# The presence of disseminated tumour cells in the bone marrow is inversely related to circulating free DNA in plasma in breast cancer dormancy

**DOI:** 10.1038/bjc.2011.537

**Published:** 2011-12-13

**Authors:** R E Payne, N L Hava, K Page, K Blighe, B Ward, M Slade, J Brown, D S Guttery, S A A Zaidi, J Stebbing, J Jacob, E Yagüe, J A Shaw, R C Coombes

**Affiliations:** 1 Division of Cancer, Imperial College, MRC Cyclotron Building, Imperial College Healthcare NHS Trust, Hammersmith Hospital, Du Cane Road, London W12 ONN, UK; 2 Department of Cancer Studies and Molecular Medicine, University of Leicester, Robert Kilpatrick Building, PO Box 65, Leicester Royal Infirmary, Leicester LE2 7LX, UK; 3 Department of Medical Oncology, Charing Cross Hospital, London W6 8RF, UK

**Keywords:** circulating tumour cells, disseminated tumour cells, circulating-free DNA, dormancy, breast cancer

## Abstract

**Background:**

The aim of this study was to gain insight into breast cancer dormancy by examining different measures of minimal residual disease (MRD) over time in relation to known prognostic factors.

**Methods:**

Sixty-four primary breast cancer patients on follow-up (a median of 8.3 years post surgery) who were disease free had sequential bone marrow aspirates and blood samples taken for the measurement of disseminated tumour cells (DTCs), circulating tumour cells (CTCs) by CellSearch and qPCR measurement of overlapping (96-bp and 291-bp) amplicons in circulating free DNA (cfDNA).

**Results:**

The presence of CTCs was correlated with the presence of DTCs measured by immunocytochemistry (*P*=0.01) but both were infrequently detected. Increasing cfDNA concentration correlated with ER, HER2 and triple-negative tumours and high tumour grade, and the 291-bp amplicon was inversely correlated with DTCs measured by *CK19* qRT-PCR (*P*=0.047).

**Conclusion:**

Our results show that breast cancer patients have evidence of MRD for many years after diagnosis despite there being no overt evidence of disease. The inverse relationship between bone marrow *CK19* mRNA and the 291-bp amplicon in cfDNA suggests that an inverse relationship between a measure of cell viability in the bone marrow (DTCs) and cell death in the plasma occurs during the dormancy phase of breast cancer.

Patients with primary breast cancer frequently have evidence of minimal residual disease (MRD) in the absence of any clinical or radiological evidence of metastases (Slade *et al*, 2009). The detection of circulating tumour cells (CTCs) in the blood and disseminated tumour cells (DTCs) in the bone marrow of disease-free patients with breast cancer has been well documented. Their presence identifies those patients with a worse prognosis, although many remain disease free for many years or decades (Meng *et al*, 2004; Braun *et al*, 2005; Slade *et al*, 2009). In addition, circulating free DNA (cfDNA) has been found in higher concentrations in cancer patients than healthy controls and has been proven to share similar genetic features to the primary tumour (Leon *et al*, 1977; Stroun *et al*, 1989). Larger fragment sizes of cfDNA, greater than the apoptotic limit, have also been detected in the blood of cancer patients; believed to be derived from tumour cell necrosis and lysis (Jahr *et al*, 2001; Wang *et al*, 2003; Umetani *et al*, 2006). The relationship of these measures of MRD to long-term breast cancer dormancy has not been fully established.

Many post-operative studies have principally been carried out by sampling patients’ bone marrow or blood shortly after surgery or adjuvant chemotherapy during the period of highest risk of relapse; however, very few studies have focused on the later ‘low risk’ period, that is, later than 4 years after surgery. Patients in this period with MRD present are likely to have cells in a dormant state although the precise mechanisms are unclear. We decided to concentrate on this period for several reasons. First, a proportion of patients relapse during this period: it is generally agreed that there is a constant rate of relapse of around 1–2% annually (van der Sangen *et al*, 2011); many patients develop evidence of widespread metastatic disease in intervals between clinic visits, and this is a considerable cause of morbidity. Second, we reasoned that if a test, or combination of tests, could be shown to indicate recurrent disease during this time, this may provide a reason to instigate systemic therapy at an earlier stage, with the aim of eradicating the disease. Finally, it was possible that we would find a subset of patients who had no evidence of disease at any time point using any test; these patients should not need to be followed up in clinic, thus reducing patient anxiety and cost.

The phenomenon of ‘dormancy’ in relation to cancer has been extensively described in the literature (Meng *et al*, 2004; Almog *et al*, 2009; Willis *et al*, 2010). The exact mechanisms of dormancy are still not clearly understood; however, evidence of a relationship between tumour dormancy and increased apoptosis exists. In a mouse model under angiogenic suppression, tumour cell proliferation was balanced by a reciprocal amount of cell death (Holmgren *et al*, 1995). We hypothesised that by combining tests indicating cell death (cfDNA) with other measures of viability (DTC and CTC detection), we could determine the relationship between the two processes.

The comparison of cfDNA with other markers of MRD has not been fully assessed, although evidence for a positive association of cfDNA with viable CTCs detected by an epithelial immunospot assay has been described (Schwarzenbach *et al*, 2009a). Conversely, no correlation of cfDNA with DTCs measured by immunocytochemistry (ICC) was found (Schwarzenbach *et al*, 2009b). In this study, we aimed to find a combination of tests to characterise dormancy mechanisms in breast cancer patients, which would enable possible routes of therapeutic intervention. For this, we compared DTCs measured by two methods (quantitative RT-PCR measurement of *cytokeratin 19* (*CK19*) and ICC, using a pan-cytokeratin antibody) and CTCs by CellSearch with two measures of cfDNA. The tests consisted of the measurement of two overlapping amplicons: a 96-bp amplicon, representing total cfDNA and a larger 291-bp amplicon that was above the apoptotic limit (185–200-bp) and believed to be mostly cancer associated. These measurements of MRD were compared with each other and with prognostic factors in patients with breast cancer on long-term follow-up.

## Materials and Methods

### Primary breast cancer patients and control groups

Three control groups were recruited to measure CTCs and cfDNA in the blood; these consisted of 34 healthy female controls (29 of which had CTCs measured) and 51 patients with benign breast disease (BBD; 28 of which had CTCs measured). Thirty-one patients with metastatic breast cancer were used as a control group to confirm raised levels of cfDNA and a higher number of CTCs (eight of these patients had CTCs measured) as compared with healthy controls (Allard *et al*, 2004; Van der Auwera *et al*, 2009). All patient groups including the primary breast cancer patients signed a written consent form; both form and protocol had been approved by the local ethics committee (RREC numbers 0975). Disseminated tumour cells in the bone marrow were not measured in the control groups for ethical reasons, but our test for bone marrow DTCs has been standardised and extensively validated in previous studies (Borgen *et al*, 1999; Slade *et al*, 1999, 2005, 2009). The amount of blood taken for CTC measurement was 22.5 ml in the healthy controls and patients with BBD and 7.5 ml in the patients with metastatic breast cancer. Twenty millilitres of blood was taken for all cfDNA analyses.

In this study, we recruited 64 patients who had been treated for primary breast cancer. Twenty-two patients had previously been recruited to our MRD studies (Slade *et al*, 2005, 2009). Twenty-nine patients were recruited from a trial of adjuvant high-dose chemotherapy (Coombes *et al*, 2005) and were selected because they had a high risk of relapse. The remaining 13 patients were selected as additional patients for statistical purposes. The inclusion criteria included a histologically confirmed breast cancer and consent to the follow-up procedure (see below). Exclusion criteria included evidence of recurrent disease on conventional staging with isotopic bone scan and computed tomography of the chest and liver/abdomen, before study entry. All patients agreed to repeat bone marrow sampling at the outset of the study followed by repeat blood sampling for further tests to measure MRD. In all, 43 (67%) patients received adjuvant chemotherapy and 58 (91%) patients received adjuvant endocrine therapy (Table 1).

### Detection of CTCs and DTCs from blood and bone marrow

The skin was incised before the bone marrow aspirates were taken to minimise the risk of epithelial contamination. Between 2 and 5 ml of bone marrow was aspirated into syringes primed with preservative-free heparin (Leo Labs, Risborough, UK). Cells were cytocentrifuged at a concentration of 5 × 10^5^ per cytospin; air-dried and stored at −20 °C before use. Slides were then stained for a common epitope of cytokeratin as previously described (Pantel *et al*, 1994; Slade *et al*, 1999; Smith *et al*, 2000). Samples that were isotype positive were deemed uninterpretable and therefore excluded from the results. The MCF-7 cell line was used as a positive control. Real-time qRT-PCR for *CK19* and *ABL* mRNA was performed as described previously (Slade *et al*, 2005). The standard used for quantification was the artificial construct in the range 10^1^–10^4^ for *CK19* and 10^3^–10^6^ for *ABL* per 2.5 *μ*l (Slade *et al*, 1999). All assays were performed with duplicate standards, a non-cDNA control and a positive cDNA control were extracted from the MCF-7 cell line. A *CK19:ABL* ratio greater or equal to 0.1% (one *CK19*/1000 *ABL* transcripts) was regarded as positive. All samples were anonymised to the person performing the assays. For the detection of CTCs, 7.5 ml of blood from the metastatic patients and 3 × 7.5 ml from the control and primary breast cancer patients, were collected in CellSave preservative tubes (Veridex, LLC, Warren, NJ, USA) from patients in London, anonymised and transported at room temperature to the Department of Surgery and Cancer, Imperial College London for processing within 72 h of collection as recommended by the manufacturer. The CellSearch System (Veridex, LLC, Warren, NJ, USA) was used for the isolation and enumeration of CTCs from each 7.5 ml of blood separately using specific morphological criteria to identify CTCs (Allard *et al*, 2004).

### CfDNA detection and analysis

Blood samples were collected in EDTA tubes and processed within 3 h of venesection. Plasma was separated by centrifugation at 1200 *g* for 10 min, and plasma was taken from the upper phase and decanted into fresh polypropylene tubes. Tubes were then spun again to remove any contaminating leucocytes (Chiu *et al*, 2001), and the resulting supernatant was aliquoted into sterile tubes and stored at −80 °C. Plasma samples were allowed to thaw to room temperature and re-spun in a bench-top centrifuge to remove any remaining cell debris as recommended (Page *et al*, 2006), and cfDNA was isolated as described previously (Page *et al*, 2011). To estimate cfDNA quantity, the concentration of a 96-bp GAPDH amplicon served as a measure of total cfDNA quantity, whereas a larger overlapping 291-bp amplicon measured total cfDNA above the limit of apoptosis. The integrity of cfDNA was calculated as a ratio of the 96-bp and 291-bp amplicons (291-bp/(291-bp+96-bp)). Primers and conditions are as described previously (Page *et al*, 2011).

### Study design

The primary objective of this prospective, longitudinal study was to investigate the natural history of DTCs in bone marrow aspirates from 64 primary breast cancer patients. This study also had secondary objectives to investigate the presence and quantity of CTCs and cfDNA in blood. Few patients relapsed with recurrent disease therefore MRD values were compared with each other and with prognostic factors in a univariate analysis. For ethical and technical reasons, samples measuring CTCs and cfDNA were taken at fewer time points as detailed below.

### Statistical methods

The main continuous variables of 96- and 291-bp amplicons in cfDNA, ICC and qRT-PCR assays for DTC, and CTC assay were positively skewed. The Spearman rank correlation (corrected for ties) was used to assess monotonic relationships between continuous variables. The Mann–Whitney test was used to compare continuous scores between two groups. One-way analysis of variance was used to compare more than two groups. A binary DTC qRT-PCR outcome was defined (<0.1% is a negative result and ⩾0.1% is a positive result).

## Results

### Patients and sampling

Sixty-four disease-free patients with a diagnosis of primary breast cancer were followed up for 1–11.5 years after surgery (a median of 8.3 years). In terms of their primary staging, 20 patients had tumours measuring <20 mm with no involved lymph nodes detected (T_1_N_0_), 4 patients had 1–3 involved lymph nodes detected, 2 patients had tumours measuring >20 mm with no involved lymph nodes detected and 38 high-risk patients had >3 involved lymph nodes at surgery. For the purposes of this study, patients were grouped into node-positive (*n*=42) or node-negative (*n*=22) groups (Table 1). Table 1 also shows patient characteristics and adjuvant systemic therapy details. The node-positive patients had bone marrow aspirates taken every 6 months, and node-negative patients every 12 months for ethical reasons. Blood samples were obtained 3–6 monthly post-operatively over a follow-up period of up to 11.5 years. For cfDNA, 2–13 (a mean of 7.4) plasma samples were analysed per patient and 0–7 (a mean of 3.5) samples for CTC analysis using the CellSearch system. For DTC analysis, 0–21 (a mean of 9.5) bone marrow aspirates were taken from each patient (both ICC and qRT-PCR if the volume was sufficient). A total of 168 bone marrow aspirates were taken at the same time points as the blood sampling and used in the direct comparison of DTCs, CTCs and cfDNA. All samples for CTC analysis also had corresponding samples for cfDNA analysis. Total measurements of DTCs, CTCs and cfDNA were used when comparing values from lymph node positive with lymph node-negative breast cancer patients.

### Minimal residual disease measurements in control groups

Only 1 of 29 (3%) healthy female controls had a single CTC detected and 4 of 28 (14%) patients with BBD had CTCs present in 22.5 ml of blood; 1 CTC was present in 3 patients and 5 CTCs were present in 1 patient. For comparative purposes, CTCs were also measured in patients with metastatic breast cancer and were present in six of eight (75%) patients (range 0–109). The quantity of both the 96- and 291-bp amplicons in cfDNA was lower in the healthy controls (*n*=34) and women with BBD (*n*=51) than in patients with metastatic breast cancer. The mean cfDNA value of the mostly cancer-associated 291-bp amplicon was 0.24 ng ml^−1^ in the healthy controls and 0.44 ng ml^−1^ in the BBD patients compared with 3.5 ng ml^−1^ in the patients with metastatic breast cancer (*P*=0.032; Table 2). The mean levels of the 291-bp cfDNA in patients with primary breast cancer were also higher than in healthy or BBD controls (3.8 ng ml^−1^). This was similar to the mean level in the metastatic patient group. Patients with primary breast cancer also showed a higher mean number of CTCs per sample compared with healthy and BBD controls (0.42 compared with 0.03 and 0.29 CTCs, respectively; Tables 2 and 3).

### Comparisons of markers of MRD with established prognostic factors in 64 breast cancer patients on long-term follow-up

Samples from multiple time points taken from each of the 64 primary breast cancer patients on long-term follow-up were included in this pooled analysis. In all, 8 of the 64 patients relapsed. There were no significant differences in cfDNA concentration, cfDNA integrity, DTC ICC or CTCs in the eight patients who had relapsed compared with the 56 who had not. However, there was a significant difference in the level of DTC detection in BM by qRT-PCR (*P*=0.025, Mann–Whitney). Next, we compared measures of MRD in all 64 patients, comparing lymph node-negative *vs* lymph node-positive patients. The mean quantities of both the 96- and 291-bp amplicons in cfDNA were correlated with the number of lymph nodes positive at surgery (*P*<0.0001 and *P*=0.0248, respectively; Table 3). However, there was extensive overlap in the value ranges between node-positive and node-negative patients (Supplementary Figure 1). Disseminated tumour cells (qRT-PCR) in the BM were positively correlated with node-positive patients (*P*=0.0154). There was no association between DTCs (ICC) or CTCs and node status. The measures of MRD (DTCs, CTCs and cfDNA) from individual blood or bone marrow samples were also compared with conventional prognostic factors in the 64 patients. In cfDNA, the 96-bp amplicon was significantly higher in patients with ER-negative (*P*=0.004), HER2-negative (*P*=0.013) and triple-negative status (*P*=0.004) compared with those whose tumours express ER or HER2. The DTC (qRT-PCR) median values were also higher in PR-negative patients (*P*=0.02) and triple-negative patients but this did not reach statistical significance (*P*=0.06; Table 4).

When considering tumour size, grade and menopausal status, there were three significant associations: the 96-bp cfDNA amplicon was significantly higher in patients with high-grade tumours (*P*=0.032), and the 291-bp cfDNA amplicon (*P*=0.033) and the number of CTCs detected (*P*=0.026) were both higher in post-menopausal women compared with pre-menopausal women (Table 5). However, there was extensive overlap in the value ranges between groups (Supplementary Figure 2).

### Relationships between different measures of MRD

In all, 41 out of the 64 patients on follow-up for primary breast cancer had both DTC and CTC values measured at the same time. Circulating tumour cells were positively correlated with DTCs measured by ICC only (*P*=0.01). This confirms our previous findings: 16 out of 19 (84%) high-risk primary breast cancer patients with DTCs present by ICC also had CTCs present (Slade *et al*, 2009). However, there was no statistically significant correlation between CTCs and DTCs measured by *CK19* qRT-PCR (Supplementary Table 1).

**Figure 1:**
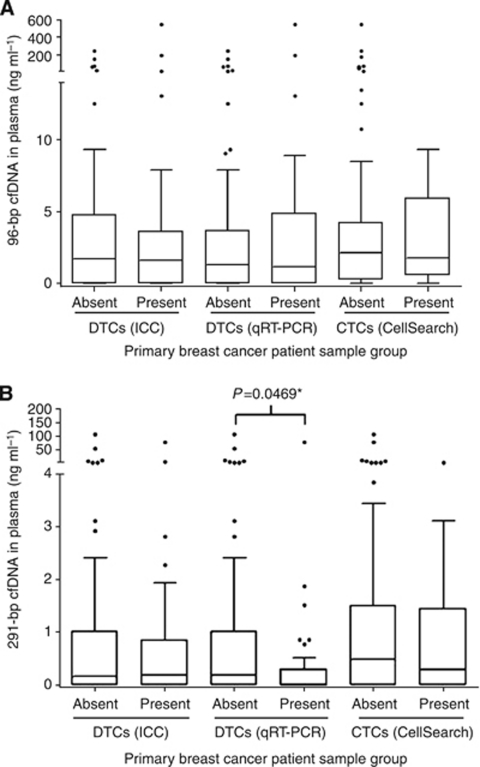
The largely cancer specific 291-bp cfDNA amplicon in plasma is inversely related to the presence of DTCs (qRT-PCR *CK19*:*ABL* ⩾0.1%) in the bone marrow of primary breast cancer patients on follow-up. Comparison of (**A**) 96-bp cfDNA and (**B**) 291-bp cfDNA in patient samples grouped by the presence or absence of DTCs (ICC and qRT-PCR) and CTCs (CellSearch) in paired bone marrow aspirate and blood samples from 64 primary breast cancer patients on follow-up. The box plots show the median and inter-quartile ranges and the whiskers indicate 1.5 × the inter-quartile range (^*^statistically significant, Mann–Whitney non-parametric *t*-test).

Next, we investigated the relationship between cfDNA with DTCs and CTCs by analysing all the matched patient samples with bone marrow aspirates and blood samples performed at the same clinic visit or within a month of either sample being taken. A total of 168 matched bone marrow and blood samples had both DTC analysis (ICC and qRT-PCR) and qPCR measurement of 96- and 291-bp amplicons in cfDNA. In all, 8 out of 64 patients had no matched samples; the rest had both a mean and median of 3 per patient. Up to 179 matched samples were analysed for both CTCs and cfDNA measuring both 96-bp and 291-bp amplicons. Interestingly, an inverse correlation was found between the presence of DTCs as measured by *CK19* qRT-PCR and the quantity of the 291-bp amplicon in cfDNA (*P*=0.0469) (Figure 1B). The box plot demonstrates that those patients with DTCs (qRT-PCR) had lower median and inter-quartile ranges of the 291-bp cfDNA amplicon than the group with no DTCs present. No other correlations were evident.

## Discussion

Patients with a high risk of relapse based on tumour stage are more likely to have bone marrow DTCs at presentation (Braun *et al*, 2005), and these patients are more likely to demonstrate CTCs during follow-up (Slade *et al*, 2009). In addition, we and others have shown that cfDNA is higher in breast cancer patients than healthy controls (Gal *et al*, 2004; Huang *et al*, 2006). It is therefore important to establish the relationship between the presence of DTCs, CTCs and cfDNA levels during long-term follow-up.

Our results show that many patients with breast cancer continue to have evidence of MRD long after the completion of their adjuvant treatment despite the fact that they have no clinically evident recurrent disease. Measures of MRD designed to detect both living and non-viable cells such as CTC/DTC (ICC) and DTC (qRT-PCR) are intermittently present; their presence does not appear to indicate inevitable relapse. Higher levels of all MRD measures were more likely in patients who had an ER-negative, HER2-negative or triple-negative tumour and who had a higher grade of tumour although only values for the 96-bp cfDNA amplicon were significantly higher. Mean quantities of both amplicons (96 and 291 bp) in cfDNA were correlated with the number of lymph nodes positive at surgery (*P*<0.0001 and *P*=0.0248, respectively). However, there was extensive overlap in the value ranges between groups for both cfDNA and the other measures of MRD suggesting that individual markers have limited prognostic value in the long-term follow-up of primary breast cancer patients (Supplementary Figures 1 and 2).

Of interest, cfDNA levels of the larger 291-bp amplicon appear inversely related to bone marrow dissemination as measured by qRT-PCR. At first glance, these results appear counter-intuitive. However, as an increase in larger sized fragments in cfDNA may result from dying micrometastases, evidence of viable DTCs as disclosed by an increase in bone marrow *CK19* mRNA by qRT-PCR may be less likely to be found in patients in whom this is happening. Immunocytochemistry is not a good measure of viable micrometastases as the method detects both viable and non-viable cells. For example, a proportion of CTCs detected by the CellSearch system have expression of a marker of apoptosis indicating they are not all viable (Rossi *et al*, 2010). Cells that are viable are also more likely to have intact mRNA and so may explain why DTCs and CTCs measured by *CK19* mRNA are a better predictive indicator of prognosis (Smith *et al*, 2000; Benoy *et al*, 2006; Farmen *et al*, 2008).

Although cfDNA is more informative, in that all blood samples contain measurable cfDNA, these results suggest that simple quantification of cfDNA alone is not a useful prognostic marker during the follow-up period and a panel of MRD markers may be required. For example, evidence that cfDNA is tumour derived using genomic technologies may provide more information about genomic alterations of clinical significance (Shaw *et al*, 2011). The fluctuations over time may be an important determinant, along with combined investigations with DTCs and CTCs to provide further information on the viability and metastatic potential of these micrometastases.

Our data raise important questions regarding the issue of dormancy in breast cancer. We and others have shown that rare DTCs and CTCs can persist for many years after the end of breast cancer treatment (Meng *et al*, 2004; Slade *et al*, 2005, 2009). As the half-life of CTCs in the blood is estimated at 1–2 h (Meng *et al*, 2004), their presence may represent a dynamic balance between proliferation from micrometastatic niches and cell death. This turnover of residual cells is possibly one of the reasons why cancer-derived cfDNA (above the apoptotic limit) persists in blood for so long after diagnosis and treatment. A mathematical model of breast cancer dormancy has recently been developed and demonstrates that long-term breast cancer dormancy can be maintained by a small subset of growth-restricted, dangerous micrometastases (Willis *et al*, 2010). This theoretical model agrees with the observations described herein of the persistence of a low but steady number of CTCs and DTCs observed from growth-restricted and not necessarily relapse-inducing micrometastases. The additional measurement and molecular characterisation of cfDNA derived from dying tumour cells may help to distinguish these phases of dormancy.

In conclusion, the inverse correlation of DTCs (by *CK19* qRT-PCR) in the bone marrow with the larger 291-bp amplicon in cfDNA indicates that the dormancy period of breast cancer is potentially characterised by two distinct phases: one in which measures of viable DTCs are absent, during which measures of cell death manifesting as cfDNA in the blood are evident, and an alternate phase, characterised by evidence of viable DTCs, but lower levels of cfDNA.

## Figures and Tables

**Table 1:** Primary breast cancer patient characteristics

	**Lymph node-positive patients**	**(%)**	**Lymph node-negative patients**	**(%)**	**Total**	**(%)**
*Menopausal status*						
Pre-menopausal	11	26	7	32	18	28
Post-menopausal	31	74	15	68	46	72
						
*Histology*						
Invasive ductal	31	74	16	73	47	73
Invasive lobular	7	17	2	9	9	14
Other/mixed invasive	4	9	4	18	8	13
						
*Tumour size*						
T1	15	36	20	91	35	55
T2	16	38	2	9	18	28
T3	7	17	0	0	7	11
T4	0	0	0	0	0	0
Unknown	4	9	0	0	4	6
						
*Tumour grade*						
I	1	2	5	23	6	9
II	20	48	9	41	29	45
III	20	48	8	36	28	44
Unknown	1	2	0	0	1	2
						
*Hormone receptor status*						
ER positive	29	69	17	77	46	72
ER negative	10	24	5	9	15	23
Unknown ER status	3	7	0	0	3	5
PgR positive	17	40	13	59	30	47
PgR negative	19	45	7	32	26	41
Unknown PgR status	6	14	2	9	8	12
						
*HER2 status*						
Positive	13	31	3	14	16	25
Negative	26	62	17	77	43	67
Unknown	3	7	2	9	5	8
						
*Endocrine therapy*						
Yes	41	98	17	77	58	91
No	1	2	5	23	6	9
						
*Chemotherapy*						
Yes	35	83	8	36	43	67
No	7	17	14	64	21	33
Total number of patients	42	66	22	34	64	100

Abbreviations: ER= oestrogen receptor; PgR= progesterone receptor.

**Table 2:** CTC measurements from blood and cell-free DNA quantity in plasma are lower in healthy controls and benign breast disease patients than in metastatic breast cancer

	**Healthy controls**	**Benign breast disease patients**	**Metastatic breast cancer patients**
*Cell-free DNA 96-bp (ng ml* ^*−1*^ *)*			
No. of patients	34	51	31
Mean	0.24	0.54	3.5
95% Confidence interval	0.09–0.63	0.27–1.07	1.48–8.29
Median	0.86	1.27	2.45
Range	0–4.52	0–8.36	0.03–1044
			
*Cell-free DNA 291-bp (ng ml* ^*−1*^ *)*			
No. of patients	34	51	31
Mean	0.24	0.44	3.5
95% Confidence interval	0–0.20	0.1–0.40	0–1.5
Median	0	0.22	0.44
Range	0–1.37	0–1.94	0–602
			
*Cell-free DNA integrity* a			
No. of patients	34	51	31
Mean	0.10	0.28	0.21
95% Confidence interval	0–0.13	0.11–0.25	0–0.34
Median	0	0.2	0.07
Range	0–0.39	0–0.49	0–0.83
			
*Circulating tumour cells* b			
No. of patients	29	28	8
Volume of blood measured	22.5 ml	22.5 ml	7.5 ml
Mean	0.03	0.29	24
95% Confidence interval	0.04–0.11	0.09–0.66	7.29–55.29
Median	0	0	9
Range	0–1	0–5	0–109
No. of patients with CTCs^c^	1 (3%)	4 (14%)	6 (75%)

Abbreviation: CTC=circulating tumour cell.

a Calculated as the ratio of the 96-bp and 291-bp amplicons (291-bp/(291-bp+96-bp)).

b Detected with the CellSearch system.

c Samples were considered positive for CTCs when at least one cell was detected per total sample volume.

**Table 3:** Minimal residual disease marker values in lymph node-positive and lymph node-negative primary breast cancer patients in samples taken over time

	**Lymph node-positive patients at surgery (*n*=42)**	**Lymph node-negative patients at surgery (*n*=22)**	***P*-value**
*Cell-free DNA 96-bp (ng ml* ^*−1*^ *)*			
Mean	19.19	14.19	<0.0001^a^
95% CI	0.61–18.93	0.24–7.52	
Median	1.06	3.01	
Range	0–2626	0–440	
No. of samples	323	152	
			
*Cell-free DNA 291-bp (ng ml* ^*−1*^ *)*			
Mean	4.35	2.66	0.0248^a^
95% CI	0.16–4.90	0.05–1.43	
Median	0.17	0.38	
Range	0–785	0–78	
No. of samples	322	149	
			
*Cell-free DNA integrity* b			
Mean	0.22	0.18	0.479
95% CI	0.001–0.029	0.001–0.037	
Median	0.17	0.13	
Range	0–1	0–1	
No. of samples	257	136	
			
*Disseminated tumour cells (qRT-PCR)* c			
Mean	0.09	0.07	0.0154^a^
95% CI	0–0.012	0.001–0.023	
Median	0.053	0.04	
Range	0–1.25	0–1.12	
No. of samples	475	133	
			
*Disseminated tumour cells (ICC)* d			
Mean	0.94	0.79	0.063
	0.005–0.15	0.009–0.28	
Median	0	0	
Range	0–15	0–12	
No. of samples	463	139	
			
*Circulating tumour cells* e,f			
Mean	0.55	0.15	0.982
	0.012–0.389	0.003–0.099	
Median	0	0	
Range	0–23	0–2	
No. of samples	153	72	

Abbreviations: CI=confidence interval; ICC=immunocytochemistry; qRT-PCR=quantitative reverse transcription-PCR.

a Statistically significant, Mann–Whitney *t*-test.

b Calculated as the ratio of the 96- and 291-bp amplicons (291-bp/(291-bp+96-bp).

c Calculated as the percentage ratio of qRT-PCR CK19:ABL.

d Detected by pan-CK antibody.

e Samples were considered positive for circulating tumour cells when at least one cell was detected in 22.5 ml of blood.

f Detected with the CellSearch system.

**Table 4:** Comparison of minimal residual disease markers in primary breast cancer patients with hormone receptor and HER2 receptor status of the primary tumour

	**ER status**		**HER2 status**		**PR status**		**Triple negative status**	
	**Positive**	**Negative**	**Positive**	**Negative**	**Positive**	**Negative**	**Yes**	**No**
*Total circulating free DNA 96-bp (ng ml* ^*−1*^ *)*								
No. of samples	341	105	128	308	220	187	81	364
Mean	21.67	4.82	21.2	15.14	9.50	30.23	5.29	20.43
95% CI	3.42 to 39.92	3.14 to 6.51	−4.81 to 47.2	−1.81 to 32.10	4.11 to 14.9	−2.54 to 63	3.15 to 7.43	3.34 to 37.53
Median	1.2	2.29	0.89	1.91	1.70	1.35	2.49	1.21
Range	0–2626	0–53.71	0–1581	0–2626	0–440	0–2626	0–53.71	0–2626
*P*-value	0.004^a^		0.013^a^		0.71		0.004^a^	
					
*Circulating free DNA 291-bp (ng ml* ^*−1*^ *)*								
No. of samples	336	106	129	305	215	188	82	360
Mean	4.46	4.46	8.08	1.79	2.08	6.85	2.84	4.21
95% CI	−0.28 to 9.20	0.79 to 3.87	−4.11 to 20.28	1.01 to 2.57	0.68 to 3.47	−1.53 to 15.22	0.86 to 4.83	−0.22 to 8.63
Median	0.23	0.19	0.1	0.28	0.38	0.16	0.24	0.23
Range	0–785.4	0–56	0–785.4	0–78.03	0–124	0–785	0–56	0–785.4
*P*-value	0.792		0.188		0.23		0.50	
				
*Disseminated tumour cells (qRT-PCR)* b								
No. of samples	440	135	171	399	273	261	106	476
Mean	0.087	0.096	0.87	0.09	0.07	0.10	0.09	0.089
95% CI	0.07–0.10	0.07–0.11	0.07–0.11	0.08–0.10	0.06–0.09	0.08–0.12	0.07–0.11	0.08–0.10
Median	0.05	0.07	0.05	0.048	0.05	0.05	0.08	0.05
Range	0–1.25	0–0.79	0–0.79	0–1.25	0–1.12	0–1.25	0–0.65	0–1.25
*P*-value	0.11		0.39		0.02^a^		0.06	
				
*Disseminated tumour cells (ICC)* c								
No. of samples	136	435	175	389	272	260	102	474
Mean	1.02	0.86	0.82	0.93	0.81	0.98	1.15	0.88
95% CI	0.67–1.36	0.72–1.00	0.60–1.03	0.75–1.11	1.47	1.82	0.70–1.60	0.74–1.02
Median	0	0	0	0	0	0	0	0
Range	0–15	0–12	0–9	0–15	0–12	0–15	0 –15	0–12
*P*-value	0.34		0.45		0.23		0.16	
				
*Circulating tumour cells* d								
No. of samples	166	48	74	135	125	72	34	178
Mean	0.36	0.67	0.54	0.38	0.40	0.51	0.91	0.34
95% CI	0.08 to 0.64	−0.15 to 1.49	−0.09 to 1.17	0.08 to 0.67	0.03 to 0.77	−0.03 to 1.06	−0.25 to 2.08	0.08 to 0.61
Median	0	0	0	0	0	0	0	0
Range	0–23	0–19	0–23	0–19	0–23	0–19	0–19	0–23
*P*-value	0.38		0.60		0.73		0.15	

Abbreviations: CI=confidence interval; ICC=immunocytochemistry; qRT-PCR=quantitative reverse transcription-PCR.

a Statistically significant difference of median values by Mann–Whitney test or mean values by *t*-test.

b Calculated as the percentage ratio of qRT-PCR *CK19:ABL* (⩾0.1 is considered positive).

c Detected by pan-CK antibody.

d Samples were considered positive for circulating tumour cells when at least one cell was detected in 22.5 ml of blood by the CellSearch system.

**Table 5:** Comparison of minimal residual disease markers in primary breast cancer patients with menopausal status and with tumour size and grade of the primary tumour

	**Menopausal status**		**Tumour size**			**Grade**		
	**Post**	**Pre**	**T1**	**T2**	**T3**	**1**	**2**	**3**
*Total circulating free DNA 96-bp (ng ml* ^*−1*^ *)*								
Mean	32.61	4.31	8.3	40.99	3.73	13.84	7.26	27.73
95% CI	3.32 to 61.9	2.6 to 6.02	3.85 to 12.74	−3.12 to 85.17	1.71 to 5.76	−9.53 to 37.21	3.46 to 11.06	−0.89 to 56.36
Median	1.72	1.72	1.91	1.01	1.12	0.55	1.27	1.9
Range	0–2626	1–197.6	0–439.6	0–2626	0–45.41	0–439.6	0–264	0–2626
No. of samples	212	262	254	139	53	38	217	2
*P*-value	0.120		0.262			0.032^a^		
								
*Circulating free DNA 291-bp (ng ml* ^*−1*^ *)*								
Mean	6.77	1.35	2.38	7.74	0.74	1.47	1.78	5.99
95% CI	−0.78 to 14.32	0.71 to 1.2	1.1 to 3.66	−3.53 to 19.08	0.2 to 1.29	0.35 to 2.53	0.39 to 3.17	−1.31 to 13.3
Median	0.32	0.095	0.4	0.14	0.12	0.03	0.17	0.29
Range	0–785.4	0–56	0–124.2	0–785.4	0–13.68	0–16.04	0–124.2	0–785.4
No. of samples	211	260	251	139	52	36	215	215
*P*-value	0.033^a^		0.102			0.305		
								
*Disseminated tumour cells(qRT-PCR)* b								
Mean	0.087	0.088	0.08	0.09	0.104	0.08	0.08	0.1
95% CI	0.07–0.1	0.07–0.1	0.07–0.1	0.07–0.11	0.07–0.14	0.05–0.1	0.07–0.10	0.08–0.11
Median	0.05	0.06	0.05	0.05	0.05	0.04	0.05	0.05
Range	0–1.12	0–1.25	0–1.124	0–1.02	0–1.25	0–0.4	0–1.25	0–1.02
No. of samples	258	358	256	192	113	49	257	290
*P*-value	0.299		0.846			0.519		
								
*Disseminated tumour cells (ICC)* c								
Mean	0.8	0.97	0.88	0.73	1.12	0.92	0.88	0.93
95% CI	0.62–0.97	0.78–1.12	0.67–1.08	0.56–0.89	0.66–1.57	0.31–1.52	0.69–1.07	0.72–1.13
Median	0	0	0	0	0	0	0	0
Range	0–11	0–15	0–12	0–7	0–15	0–12	0–9	0–15
No. of samples	259	351	259	215	87	47	262	284
*P*-value	0.307		0.378			0.809		
								
*Circulating tumour cells* d								
Mean	0.37	0.46	0.17	0.59	1.03	0.35	0.15	0.68
95% CI	−0.09 to 0.83	0.14 to 0.79	0.09 to 0.25	−0.06 to 1.25	−0.34 to 2.4	0.01 to 0.68	0.07 to 0.23	0.12 to 1.24
Median	0	0	0	0	0	0	0	0
Range	0–23	0–19	0–2	0–23	0–19	0–3	0–2	0–23
No. of samples	100	125	120	71	29	23	95	107
*P*-value	0.026^a^		0.197			0.201		

Abbreviations: CI=confidence interval; ICC=immunocytochemistry; qRT-PCR=quantitative reverse transcription-PCR.

a Statistically significant by one-way analysis of variance, Kruskal–Wallis or Mann–Whitney *t*-test.

b Calculated as the percentage ratio of qRT-PCR *CK19:ABL* (0.1 or more is considered positive).

c Detected by pan-CK antibody.

d Samples were considered positive for circulating tumour cells when at least one cell was detected in 22.5 ml of blood by the CellSearch system.

## References

[bib1] Allard WJ, Matera J, Miller MC, Repollet M, Connelly MC, Rao C, Tibbe AG, Uhr JW, Terstappen LW (2004) Tumor cells circulate in the peripheral blood of all major carcinomas but not in healthy subjects or patients with nonmalignant diseases. Clin Cancer Res 10: 6897–6904 15501967 10.1158/1078-0432.CCR-04-0378

[bib2] Almog N, Ma L, Raychowdhury R, Schwager C, Erber R, Short S, Hlatky L, Vajkoczy P, Huber PE, Folkman J, Abdollahi A (2009) Transcriptional switch of dormant tumors to fast-growing angiogenic phenotype. Cancer Res 69: 836–844 19176381 10.1158/0008-5472.CAN-08-2590

[bib3] Benoy IH, Elst H, Philips M, Wuyts H, Van Dam P, Scharpe S, Van Marck E, Vermeulen PB, Dirix LY (2006) Prognostic significance of disseminated tumor cells as detected by quantitative real-time reverse-transcriptase polymerase chain reaction in patients with breast cancer. Clin Breast Cancer 7: 146–152 16800974 10.3816/CBC.2006.n.024

[bib4] Borgen E, Naume B, Nesland JM, Kvalheim G, Beiske K, Fodstad O, Diel I, Solomayer EF, Theocharous P, Coombes RC, Smith BM, Wunder E, Marolleau JP, Garcia J, Pantel K (1999) Standardization of the immunocytochemical detection of cancer cells in BM and blood: I. Establishment of objective criteria for the evaluation of immunostained cells. Cytotherapy 1: 377–388 20426539 10.1080/0032472031000141283

[bib5] Braun S, Vogl FD, Naume B, Janni W, Osborne MP, Coombes RC, Schlimok G, Diel IJ, Gerber B, Gebauer G, Pierga JY, Marth C, Oruzio D, Wiedswang G, Solomayer EF, Kundt G, Strobl B, Fehm T, Wong GY, Bliss J, Vincent-Salomon A, Pantel K (2005) A pooled analysis of bone marrow micrometastasis in breast cancer. N Engl J Med 353: 793–802 16120859 10.1056/NEJMoa050434

[bib6] Chiu RW, Poon LL, Lau TK, Leung TN, Wong EM, Lo YM (2001) Effects of blood-processing protocols on fetal and total DNA quantification in maternal plasma. Clin Chem 47: 1607–1613 11514393

[bib7] Coombes RC, Howell A, Emson M, Peckitt C, Gallagher C, Bengala C, Tres A, Welch R, Lawton P, Rubens R, Woods E, Haviland J, Vigushin D, Kanfer E, Bliss JM (2005) High dose chemotherapy and autologous stem cell transplantation as adjuvant therapy for primary breast cancer patients with four or more lymph nodes involved: long-term results of an international randomised trial. Ann Oncol 16: 726–734 15817602 10.1093/annonc/mdi166

[bib8] Farmen RK, Nordgard O, Gilje B, Shammas FV, Kvaloy JT, Oltedal S, Heikkila R (2008) Bone marrow cytokeratin 19 mRNA level is an independent predictor of relapse-free survival in operable breast cancer patients. Breast Cancer Res Treat 108: 251–258 17492378 10.1007/s10549-007-9592-x

[bib9] Gal S, Fidler C, Lo YM, Taylor M, Han C, Moore J, Harris AL, Wainscoat JS (2004) Quantitation of circulating DNA in the serum of breast cancer patients by real-time PCR. Br J Cancer 90: 1211–1215 15026803 10.1038/sj.bjc.6601609 PMC2409649

[bib10] Holmgren L, O’Reilly MS, Folkman J (1995) Dormancy of micrometastases: balanced proliferation and apoptosis in the presence of angiogenesis suppression. Nat Med 1: 149–153 7585012 10.1038/nm0295-149

[bib11] Huang ZH, Li LH, Hua D (2006) Quantitative analysis of plasma circulating DNA at diagnosis and during follow-up of breast cancer patients. Cancer Lett 243: 64–70 16412565 10.1016/j.canlet.2005.11.027

[bib12] Jahr S, Hentze H, Englisch S, Hardt D, Fackelmayer FO, Hesch RD, Knippers R (2001) DNA fragments in the blood plasma of cancer patients: quantitations and evidence for their origin from apoptotic and necrotic cells. Cancer Res 61: 1659–1665 11245480

[bib13] Leon SA, Shapiro B, Sklaroff DM, Yaros MJ (1977) Free DNA in the serum of cancer patients and the effect of therapy. Cancer Res 37: 646–650 837366

[bib14] Meng S, Tripathy D, Frenkel EP, Shete S, Naftalis EZ, Huth JF, Beitsch PD, Leitch M, Hoover S, Euhus D, Haley B, Morrison L, Fleming TP, Herlyn D, Terstappen LW, Fehm T, Tucker TF, Lane N, Wang J, Uhr JW (2004) Circulating tumor cells in patients with breast cancer dormancy. Clin Cancer Res 10: 8152–8162 15623589 10.1158/1078-0432.CCR-04-1110

[bib15] Page K, Hava N, Ward B, Brown J, Guttery DS, Ruangpratheep C, Blighe K, Sharma A, Walker RA, Coombes RC, Shaw JA (2011) Detection of HER2 amplification in circulating free DNA in patients with breast cancer. Br J Cancer 104: 1342–1348 21427727 10.1038/bjc.2011.89 PMC3078598

[bib16] Page K, Powles T, Slade MJ, MT DEB, Walker RA, Coombes RC, Shaw JA (2006) The importance of careful blood processing in isolation of cell-free DNA. Ann NY Acad Sci 1075: 313–317 17108226 10.1196/annals.1368.042

[bib17] Pantel K, Felber E, Schlimok G (1994) Detection and characterization of residual disease in breast cancer. J Hematother 3: 315–322 7735827 10.1089/scd.1.1994.3.315

[bib18] Rossi E, Basso U, Celadin R, Zilio F, Pucciarelli S, Aieta M, Barile C, Sava T, Bonciarelli G, Tumolo S, Ghiotto C, Magro C, Jirillo A, Indraccolo S, Amadori A, Zamarchi R (2010) M30 neoepitope expression in epithelial cancer: quantification of apoptosis in circulating tumor cells by CellSearch analysis. Clin Cancer Res 16: 5233–5243 20978147 10.1158/1078-0432.CCR-10-1449

[bib19] Schwarzenbach H, Alix-Panabieres C, Muller I, Letang N, Vendrell JP, Rebillard X, Pantel K (2009a) Cell-free tumor DNA in blood plasma as a marker for circulating tumor cells in prostate cancer. Clin Cancer Res 15: 1032–1038 19188176 10.1158/1078-0432.CCR-08-1910

[bib20] Schwarzenbach H, Pantel K, Kemper B, Beeger C, Otterbach F, Kimmig R, Kasimir-Bauer S (2009b) Comparative evaluation of cell-free tumor DNA in blood and disseminated tumor cells in bone marrow of patients with primary breast cancer. Breast Cancer Res 11: R71 19772563 10.1186/bcr2404 PMC2790848

[bib21] Shaw JA, Page K, Blighe K, Hava N, Guttery D, Ward B, Brown J, Ruangpratheep C, Stebbing J, Payne R, Palmieri C, Cleator S, Walker RA, Coombes RC (2011) Genomic analysis of circulating cell free DNA infers breast cancer dormancy. Genome Res; e-pub ahead of print 11 October 2011 10.1101/gr.123497.111 PMC3266030 21990379

[bib22] Slade MJ, Payne R, Riethdorf S, Ward B, Zaidi SA, Stebbing J, Palmieri C, Sinnett HD, Kulinskaya E, Pitfield T, McCormack RT, Pantel K, Coombes RC (2009) Comparison of bone marrow, disseminated tumour cells and blood-circulating tumour cells in breast cancer patients after primary treatment. Br J Cancer 100: 160–166 19034279 10.1038/sj.bjc.6604773 PMC2634698

[bib23] Slade MJ, Singh A, Smith BM, Tripuraneni G, Hall E, Peckitt C, Fox S, Graham H, Luchtenborg M, Sinnett HD, Cross NC, Coombes RC (2005) Persistence of bone marrow micrometastases in patients receiving adjuvant therapy for breast cancer: results at 4 years. Int J Cancer 114: 94–100 15523696 10.1002/ijc.20655

[bib24] Slade MJ, Smith BM, Sinnett HD, Cross NC, Coombes RC (1999) Quantitative polymerase chain reaction for the detection of micrometastases in patients with breast cancer. J Clin Oncol 17: 870–879 10071278 10.1200/JCO.1999.17.3.870

[bib25] Smith BM, Slade MJ, English J, Graham H, Luchtenborg M, Sinnett HD, Cross NC, Coombes RC (2000) Response of circulating tumor cells to systemic therapy in patients with metastatic breast cancer: comparison of quantitative polymerase chain reaction and immunocytochemical techniques. J Clin Oncol 18: 1432–1439 10735890 10.1200/JCO.2000.18.7.1432

[bib26] Stroun M, Anker P, Maurice P, Lyautey J, Lederrey C, Beljanski M (1989) Neoplastic characteristics of the DNA found in the plasma of cancer patients. Oncology 46: 318–322 2779946 10.1159/000226740

[bib27] Umetani N, Giuliano AE, Hiramatsu SH, Amersi F, Nakagawa T, Martino S, Hoon DS (2006) Prediction of breast tumor progression by integrity of free circulating DNA in serum. J Clin Oncol 24: 4270–4276 16963729 10.1200/JCO.2006.05.9493

[bib28] Van der Auwera I, Elst HJ, Van Laere SJ, Maes H, Huget P, van Dam P, Van Marck EA, Vermeulen PB, Dirix LY (2009) The presence of circulating total DNA and methylated genes is associated with circulating tumour cells in blood from breast cancer patients. Br J Cancer 100: 1277–1286 19367284 10.1038/sj.bjc.6605013 PMC2676551

[bib29] van der Sangen MJ, van de Wiel FM, Poortmans PM, Tjan-Heijnen VC, Nieuwenhuijzen GA, Roumen RM, Ernst MF, Tutein Nolthenius-Puylaert MC, Voogd AC (2011) Are breast conservation and mastectomy equally effective in the treatment of young women with early breast cancer? Long-term results of a population-based cohort of 1451 patients aged </=40 years. Breast Cancer Res Treat 127: 207–215 20703938 10.1007/s10549-010-1110-x

[bib30] Wang BG, Huang HY, Chen YC, Bristow RE, Kassauei K, Cheng CC, Roden R, Sokoll LJ, Chan DW, Shih Ie M (2003) Increased plasma DNA integrity in cancer patients. Cancer Res 63: 3966–3968 12873992

[bib31] Willis L, Alarcon T, Elia G, Jones JL, Wright NA, Tomlinson IP, Graham TA, Page KM (2010) Breast cancer dormancy can be maintained by small numbers of micrometastases. Cancer Res 70: 4310–4317 20501854 10.1158/0008-5472.CAN-09-3144

