# Supplementary figures and images for: The presence of disseminated tumour cells in the bone marrow is inversely related to circulating free DNA in plasma in breast cancer dormancy
Source: Br J Cancer. 2011 Dec 13;106(2):375–82. doi: 10.1038/bjc.2011.537 (PMC3261674; doi:10.1038/bjc.2011.537)

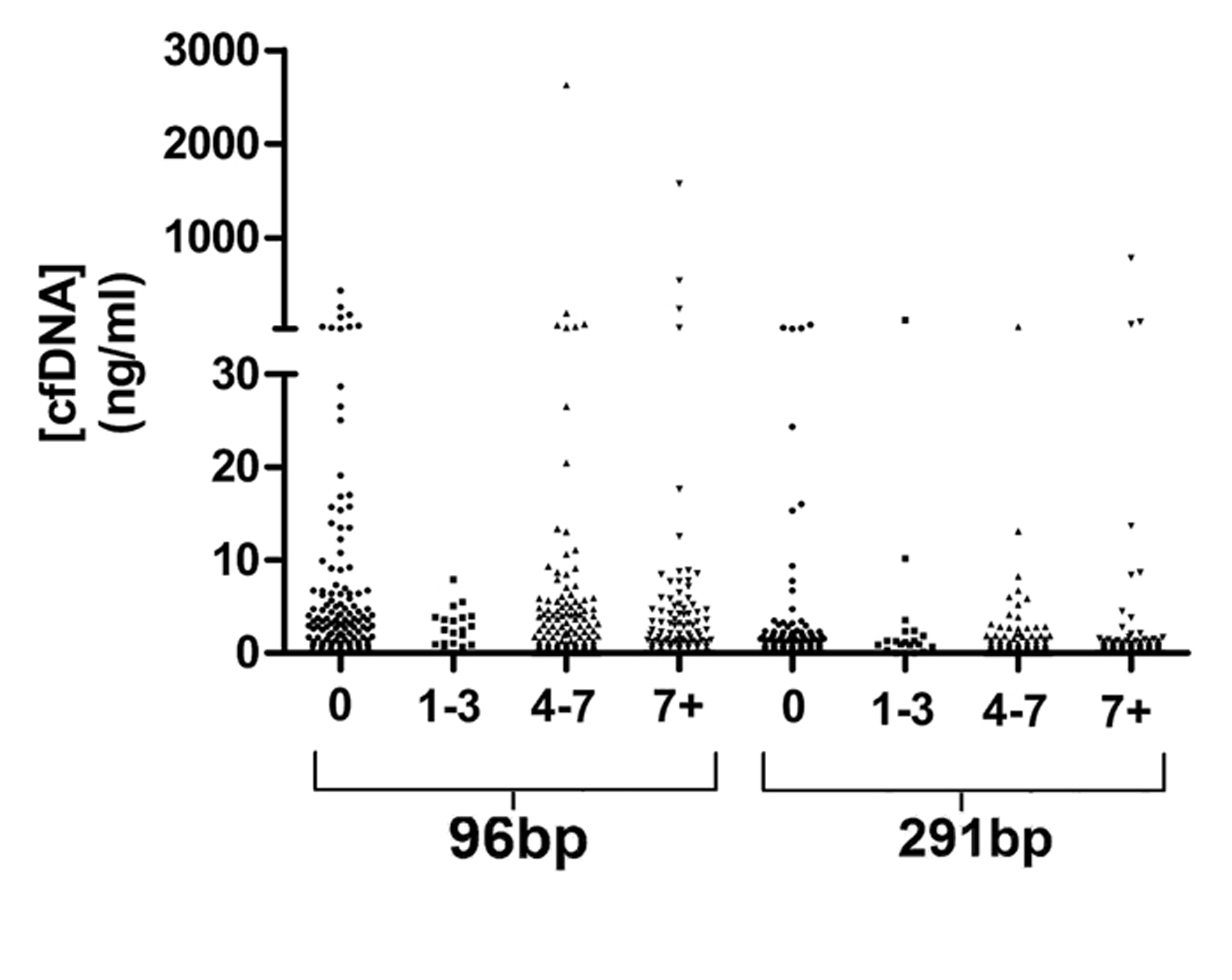

Supplement: Supplementary Figure 1 [file bjc2011537x1.tif]

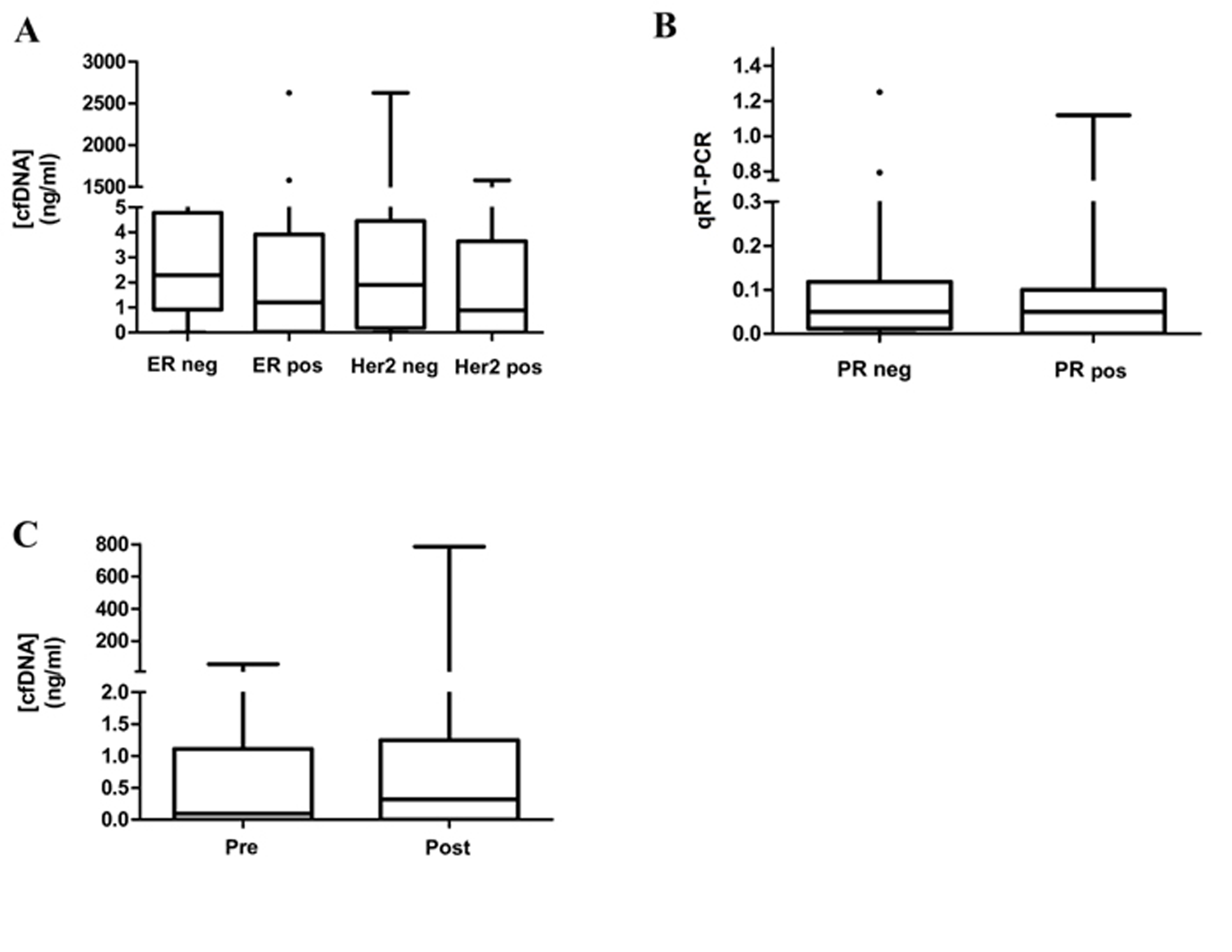

Supplement: Supplementary Figure 2 [file bjc2011537x2.tif]
