# Supplementary material for: The presence of disseminated tumour cells in the bone marrow is inversely related to circulating free DNA in plasma in breast cancer dormancy
Source: Br J Cancer. 2011 Dec 13;106(2):375–82. doi: 10.1038/bjc.2011.537 (PMC3261674; doi:10.1038/bjc.2011.537)
Supplement: Supplementary Figure Legends [file bjc2011537x3.doc]

**New Supplementary Figure 1.** Scatter plot showing quantity of 96bp and 291bp amplicons in cfDNA, by number of positive lymph nodes.

**New Supplementary Figure 2.** Box and whisker plots showing cfDNA quantity by ER status, HER statusand menopausal status, and DTC (qRT-PCR) by PR status. There was extensive overlap in the value ranges between groups for both cfDNA and the other measures of MRD suggesting that individual markers have limited prognostic value in the long term follow up of primary breast cancer patients
